# Nutritional and sensory properties of low‐fat milk dessert enriched with quinoa (*Chenopodium quinoa* Willd) Titicaca protein isolate

**DOI:** 10.1002/fsn3.3082

**Published:** 2022-09-28

**Authors:** Seyed Saeed Sekhavatizadeh, Abdolhamid Karimi, Saeid Hosseinzadeh, Amir Reza Shaviklo, Mohsen Abedi, Hamidreza Mahmoodianfard, Mohsen Ghaedmohammadi

**Affiliations:** ^1^ Assistant Professor of Fars Agricultural and Natural Resources Research and Education Center AREEO Shiraz Fars Iran; ^2^ Assistant Professor of Animal Science Research, Fars Agricultural and Natural Resources Research and Education Center, Agricultural Research Education and Extension Organization AREEO Shiraz Iran; ^3^ Professor of Food Hygiene, Department of Food Hygiene and Public Health, School of Veterinary Medicine Shiraz University Shiraz Iran; ^4^ Associate Professor of Food Science and Technology, Department of Animal Processing, Animal Science Research Institute of Iran Agricultural Research, Education and Extension Organization (AREEO) Karaj Iran; ^5^ Lecturer of Agricultural Education and Extension Institute, Agricultural Research, Education and Extension Organization (AREEO) Tehran Iran; ^6^ Lecturer of Fars Agricultural and Natural Resources Research and Education Center AREEO Shiraz Iran

**Keywords:** amino acid profile, enrichment, Milk dessert, quinoa protein, Titicaca

## Abstract

The purpose of this work was to investigate the potential production of *Titicaca quinoa* protein isolated (TQPI) to improve the quality of low‐fat desserts. In this study, low‐fat desserts incorporating TQPI (0, 1%, 3%, and 5%) were produced. The results indicated that as TQPI increased, protein content, acidity, b*, hardness, and water‐holding capacity (WHC) increased. Dessert containing 5% TQPI exhibited the highest values of hardness (63.23 ± 1.46 g), adhesiveness (0.88 ± 0.19), gumminess (67.30 ± 1.41 g), chewiness (11.41 ± 0.46 mJ), protein content (18.09%), b*(20.75), WHC (50.65%), and acidity (25.9 °D) on the 21st day of the storage time. TQPI (1%) gave a better effect on taste, texture, and total acceptability in comparison with other fortified desserts. Electron microscopy shows that the fortified dessert containing 5% TQPI had a stronger network than the others. It can be concluded that desserts containing 1% TQPI presented a very good response as a potential new dairy product based on sensory properties.

## Graphical abstract

The dessert containing 1% TQPI presented suitable physicochemical and sensory properties. The dessert containing 5% TQPI exhibited the highest values of texture parameters, protein content and WHC at 21st storage time.

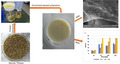

## 1 INTRODUCTION

Desserts are well known in many cultures of the society as sweet courses that typically come at the end of a meal. Due to serious health concerns, the food industry is developing a variety of foods with improved properties and tastes for consumer health and well‐being, while eating fat, sugar, salt, and certain pressure is being applied to reduce the number of additives (Jahromi & Niakousari, 2018). One of the fat‐substituted materials is protein. Studies on the effects of protein fortification and their concentration on dessert properties such as texture, appearance, physical and sensory properties during the storage are of increasing interest (Faturrahman et al., 2015; Grek et al., 2018; Komatsu et al., 2013; Levin et al., 2016; Nepovinnykh et al., 2019; Nunes et al., 2003; Pracham & Thaiudom, 2016).

The highly nutritious grain, Quinoa (*Chenopodium quinoa* Willd.), is usually cultivated in the Andean highlands of Peru, Bolivia, Ecuador, Chile, Argentina, and Colombia. Among the genera, Titicaca is the species with the highest yield (4.48 t/ha) and the largest cultivation areas in Iran (Bazile et al., 2016). The most notable properties of quinoa grains are their nutritional, phenol, and polyphenol content (Kibar et al., 2021). The gluten protein is absent in quinoa, while it contains plenty of polyunsaturated fats and high dietary fiber, which can be categorized as a low glycemic index food. Quinoa is the most beneficial food for lactose intolerance, diabetes, and hyperlipidemia patients (Cao et al., 2021). Quinoa has recently been nominated as a new protein resource diet with a high protein content and balanced amino acid. Moreover, the grain contains higher levels of lysine (5.1%–6.4%), methionine (0.4%–1.0%), and cysteine compared to other regular grains (Shen et al., 2021). The main proteins of quinoa are 2S albumin and 11S globulin which make up about 35% and 37% of the total cereal protein, respectively (Kaspchak et al., 2017).

The quinoa protein isolate (QPI) has excellent technological properties like water–oil binding, foaming, emulsifying, solubility, and gelation properties (Shen et al., 2021). Kaspchak et al. (2017) showed that when the QPI is heated to 70–90°C at pH 3.5, a strong and stable gel is formed, and the possibility of gel formation is affected by the pH value through changes in secondary structure and protein solubility. An improved water–oil‐binding capacity of QPI compared to some legume proteins was reported by Steffolani et al. (2016), but its properties vary depending on the genotype of quinoa. The technological properties of proteins depend on many factors, including charge, density, water activity, temperature, hydrophilic/hydrophobic ratios, ionic power, pH, and environmental changes (Abugoch et al., 2008). The technological properties of proteins vary depending on the processing conditions. The maximum solubility of QPI was reached at alkaline pH (10). The QPI is a source of nutrients that make it suitable as a dietary supplement in functional food (Ghumman et al., 2021). The protein extraction is positively influenced by the stirring time. The maximum extractability power of protein was noted at 120 min. However, limited research has been conducted on the use of QPI in food products. But the results of these studies have been satisfied, for example in the research of Gupta et al. (2021) who reported pasta with QPI had a potential option for high‐quality low‐cost products with enhanced nutritional and technological properties (Gupta et al., 2021). Therefore, these findings led us to study the possibility of using *Titicaca quinoa* protein isolated (TQPI) to create a new milk‐based dessert and for the determination of its nutritional and technological properties. The objectives of the present paper were to study the effects of adding different concentrations of TQPI on the microstructural, chemical, and physical properties of low‐fat desserts.

## 2 MATERIALS AND METHODS

### 2.1 Materials

Sulfuric acid, boric acid, methyl red, chloroform, methanol, hexane, hydrochloric acid, NaOH, KH_2_PO_4_, standards including methanol (HPLC grade) sodium acetate, methanol, borate buffer, sodium hydroxide, and hydrochloric acid were purchased from Merck. Norovalin, o‐phthalaldehyde (OPA), and 2‐mercaptoethanol (2ME) were purchased from Sigma Chemical Co. The milk that contained 8.1% nonfat dry matter, 1.5% fat, 3.2% protein, pH 6.6, acidity 16 °D, and density 1.035 g/cm^2^ was obtained from Pegah Fars Dairy factory, Shiraz, Fars, Iran. The quinoa with dry matter 25.95%, protein 17.52%, fat 3.9%, ash 3.67%, and carbohydrate 74.91% was purchased from Laserboresh Co.

### 2.2 TQPI production

To remove lipids from the sample, chloroform:methanol (2:1), 1:10 w/v with shaking for 2 h was employed. This procedure was repeated three times. Quinoa protein was prepared based on the method of Elsohaimy et al. (2015). Briefly, defatted quinoa flour (50 g) was suspended in 1000 ml of deionized distilled water (1:20 v/v), and the pH was adjusted to 11 using 0.1 N NaOH. To obtain the maximum degree of solubilization, the pH was kept constant after stirring the suspension for 24 h. The mixture was centrifuged at 6000 *g* for 30 min at 20°C through a high‐speed refrigerated centrifuge (Sigma 3‐16pk, Sigma). Then HCl 0.1 N was used for adjusting the pH of the supernatant to 4.5. The suspension was centrifuged at 10,000 *g* for 45 min at 4°C. Then, it was washed with distilled water. The precipitate was collected, lyophilized, and stored at 20°C for further use (Elsohaimy et al., 2015).

### 2.3 Dessert preparation

Four equal parts of the dessert were prepared by the reduced‐fat milk (1.5% w/w fat). The corresponding milk powder was dissolved in 0.1 mol L^−1^ NaCl solution to gain a final concentration of 10% w/w and stirred at 300 rpm (revolutions per minute) (magnetic stirrer). To prepare the samples, dry blended sucrose, modified starch, and (1, 3, and 5% w/w) of TQPI were added to desserts separately. The mixture was then added to the rehydrated milk, before being stirred at room temperature for 30 min. The supplemented milk samples consisted of TQPI (1, 3, and 5) % and homogenized at 65°C at 100–150 kg∙cm^−2^ for 5 min in a homogenizer. A nonfortified dessert was employed as the control. Then they were heated at 75°C (for 10 min). The final composition of the dessert (in 100 g of the product) was as follows: 8 g sucrose and 2 g modified starch. The samples were then mixed for an extra 5 min at 50°C. All treatments were packed in cups (100 g) at 80°C. Heat seal aluminum foil lids were applied for sealing the dessert cups. They were stored at 4°C in the refrigerator. Chemical parameters including titratable acidity, WHC, pH, color, and sensory properties were evaluated during the storage at 7 days intervals for 21 days. The aim of this study was to evaluate the nutritional and chemical properties of the product, therefore, the electron microscopy and texture analysis were performed on the first and last days of experiments (Karimi et al., 2021).

### 2.4 Proximate value of TQPI and dessert

Samples of TQPI and desserts were analyzed for moisture, energy levels, and major nutrient content (fat, ash, protein, and carbohydrates) using the method described by the Association of Official Analytical Chemists (AOAC) on the first day of storage (Horwitz, 2010). The Macro‐Kjeldahl method (6.25 for quinoa flour) (KjelFlex K360, Büchi, Flawil, Switzerland) was used for protein measurements. A Soxhlet extractor was used to measure crude fat. Moreover, ash was determined based on burning at 550 ± 15°C (Mariotti et al., 2008). The following Equations (1) and (2) were used to calculate the total carbohydrate and energy values (Bazile et al., 2016) and (Cardoso et al., 2019):

(1) $$\text{Total carbohydrates}\;\left(g/100\;g\right)=100-\left(m\;\mathrm{fat}+m\;\mathrm{ash}+m\;\text{proteins}\right)$$

(2) $$\text{Energy}\;\left(\text{kcal}/100\;g\right)=4\times \left(m\;\text{proteins}+m\;\text{carbohydrates}\right)+9\times \left(m\;\mathrm{fat}\right)$$

### 2.5 Titratable acidity and pH

A pH meter (Greisinger electronic, Germany) was used to record the pH of the controls and TQPI desserts. The Dornic Grade (Iranian National Standard, 2852) was employed to measure the titrable acidity of the samples.

### 2.6 Water‐holding capacity

The WHC was determined according to the method described by Remeuf et al. (2003). Approximately 20 g of dessert sample (New York (NY)) was centrifuged for 10 min at 483 × g and 20°C. The whey expelled was excluded and weighed (Cao et al.). The WHC was calculated based on the following Equation 3, (Remeuf et al., 2003).

(3) $$\mathrm{WHC}\;\left(\%\right)=100\times \left(\mathrm{NY}-\text{WE}\right)/\mathrm{NY}$$

### 2.7 Amino acid profile analysis of dessert

The following reagents were used to derive the amino acids: 0.01 M sodium acetate in water (mobile phase A) and methanol (mobile phase B). The borate buffer (0.5 M; pH: 10.2); for the preparation of reagent 2 ME / OPA (pH: 9.3): 0.05 g of the OPA was dissolved in 500 μl of borate buffer (pH: 9.9) with 4.5 μl of methanol, 25 μl of 2ME, and norovalin reagent: 50 μl of norovalin)concentration 1000 μM) was added to 950 μl of 0.01 M HCl. Amino acid analysis was performed after the seed sample was hydrolyzed to 6 mol. L^−1^ HCl and 0.5 g. L^−1^ of β‐mercaptoethanol in a tube vacuum sealed at 110°C for 21 h. After cooling, the hydrolysate was centrifuged at 6000 x *g* and submitted for 30 min, and the precipitates were discarded. The sample was neutralized with 3.0 M NaOH. A successive sampling of 100 μl of borate buffer and 25 μl of the sample was then mixed twice for 0.5 min. Then 50 μl of the 2ME/OPA reagent was added. After mixing six times, 100 μl 0.1 N HCl was collected and mixed six times. Then, 50 μl of this solution was added to 200 μl of mobile phase A, mixed twice, and finally, 20 μl of the mixture was injected. The 10 μl injection volume was used for the samples. Separation was performed using a 4 mm x 25 cm guard column (ProntoSIL (PS) Spheribond 80‐5 ODS 2), AK351, Eclipse AAA (4.6 x 15 mm); particle size: 5 μm, 40°C, with a flow rate of 1.0 ml.min^−1^ on Agilent 1100 HPLC series, USA, fluorescence detector (FLD) equipped (excitation: 450 nm, emission: 348 nm). The amino acid content was given in mg/100 gd. m (Sekhavatizadeh et al., 2021). The amino acid profile analysis was performed on the first day of the storage.

### 2.8 Color parameters

Changes in dessert color were measured by the use of a Chroma Meter CR‐400 (Japan). L*, a*, and b* values were expressed as L* (black to white), a* (green to red), and b* (blue to yellow) (Rahpeyma & Sekhavatizadeh, 2020).

### 2.9 Texture analysis

The texture analysis [Brookfield CT3 4500 (USA)] of the dessert was performed on the first and the last days of the preparation. A TA11/1000 cylindrical probe was employed to perform the texture profile analysis (TPA). The height and diameter of the samples were 30 mm and 20 mm, respectively. The penetration of 20 mm was determined at the following rates: 1 mm/s before the test, 1 mm/s in the test, and 10 mm/s after the test. Texture parameters were obtained from the device including hardness (average force of first and second stages) (g), cohesiveness, chewiness (mJ), gumminess (g), and adhesiveness (mJ). For each sample, all of these measurements were repeated at least 3 times at a temperature of 25 ± 3°C (Dokoohaki et al., 2019).

### 2.10 SEM

The lyophilized sample was fixed in an aluminum holder and covered with gold with a sputter coater (Desk Sputter Coater DSR1, Nanostructured Coatings Co.) before inspection with a scanning electron microscope (SEM, TESCAN, VEGA3, Czech Republic). Next, the sample was observed under an acceleration voltage of 10.0 kV. The working distance between the microscope objective and the sample surface ranged from 7.03 to 8.91 mm (Karimi et al., 2021).

### 2.11 Sensory properties

The dessert sensory evaluation was performed by 30 trained panel participants composed of students and laboratory staff. The age composition of the panelists was 70% (22–37) and 30% (38–50), whereas the gender composition was 40% male and 60% female. The methodology was based on a hedonic 5‐point scale (5 = very like, 1 = very disliked). Quality attributes such as color, odor, taste, overall acceptability, and texture were evaluated. Panelists felt free to describe the formulations. Green/grassy and sandy or coarse texture was reported by the panelist. The presence of granules that remained intact after oral digestion was considered a sandiness sensation. Freshly cut grass was considered for grassy substance reference. Twenty grams of dessert samples was then offered to each expert panelist under white fluorescent light. Samples were presented in the same location as used for the sensory panel and in a similar manner regarding lighting, containers, rinsing water, sample codification, and presentation order. The samples were served for sensory assessment and consumers were asked to evaluate the sample without any break. Hedonic ratings are 1 for the lowest score and 5 for the highest score (Chiavaro et al., 2011; Gunness et al., 2009; Karimi et al., 2021; Laguna et al., 2021 & Soukoulis et al., 2010).

### 2.12 Statistical analysis

Data were analyzed with SPSS version 21.0. Titratable acidity, pH, WHC, texture, color, and sensory properties were analyzed by two‐way analysis of variance (ANOVA) with a confidence level (CI) of 0.05 to determine the presence or absence of significant differences between TQPI % and time factors. Dry matter, protein, fat, ash, carbohydrate, and energy values were analyzed by one‐way ANOVA. The means were compared with Duncan’s multiple range tests and then employed as a post hoc test at a significance level of 0.05. All experiments were completed in triplicate, except for the amino acid profile.

## 3 RESULTS AND DISCUSSIONS

### 3.1 Proximate value of TQPI and dessert

The physicochemical properties of TQPI and desserts were enhanced at various TQPI levels (Table 1). The addition of TQPI increased the protein content of the dessert from (13.62 + 0.17) % in the control sample without TQPI to (18.09 + 0.13) % with the addition of TQPI (5% w/w) to the dessert formula. Conversely, the fat content of TQPI‐rich desserts was significantly lower compared to the control desserts (*p* ˂ .05). Samples supplemented with TQPI showed higher dry matter content compared to the control desserts. Previous studies have shown similar results with the addition of cobia, flaxseed, and lupin powder to desserts (Abugoch et al., 2008).

**TABLE 1:** Proximate value of TQPI and fortified dessert

Parameters	TQPI	Fortified dessert			
		Control	1%	3%	5%
Dry matter^c^	98.12 ± 0.27	25.90 ± 0.26d	26.74 ± 0.13c	28.22 ± 0.24b	29.56 ± 0.42a
Protein^d^	87.80 ± 1.61	13.62 ± 0.17d	14.33 ± 0.11c	15.57 ± 0.20b	18.09 ± 0.13a
Fat^d^	0.63 ± 0.01	6.40 ± 0.08a	6.21 ± 0.07ab	6.08 ± 0.11c	5.94 ± 0.15c
Ash^d^	2.13 ± 0.6	2.62 ± 0.1a	2.60 ± 0.09a	2.54 ± 0.12a	2.43 ± 0.10a
Carbohydrates^a^	9.22 ± 1.80	77.36 ± 0.18a	76.86 ± 0.27a	75.80 ± 0.42b	73.53 ± 0.37c
Energy^b^ ^,^ ^e^	390.37 ± 0.30	421.51 ± 0.78a	420.68 ± 0.04ab	420.23 ± 0.07b	419.98 ± 0.72b
pH _(in 1 day)_	4.84 ± 0.03	6.68 ± 0.24aA	6.45 ± 0.05abA	6.18 ± 0.05cA	5.93 ± 0.17cA
pH _(in 7 day)_		6.30 ± 0.15aB	6.20 ± 0.07aB	5.90 ± 0.08bA	5.75 ± 0.14bAB
pH _(in 14 day)_		6.25 ± 0.23aB	6.00 ± 0.22aBC	5.83 ± 0.22aA	5.83 ± 0.18aA
pH _(in 21 day)_		6.18 ± 0.12aB	5.90 ± 0.11abC	5.76 ± 0.28bcA	5.42 ± 0.2cB
Acidity _(in 1 day)_	16.33 ± 0.53	16.30 ± 1.57aD	16.20 ± 1.31aB	15.70 ± 1.47aB	15.90 ± 2.15aC
Acidity _(in 7 day)_		21.00 ± 1.00aC	21.40 ± 1.44aB	21.60 ± 0.53aAB	21.90 ± 1.82aB
Acidity _(in 14 day)_		22.90 ± 1.15aAB	23.10 ± 1.15aA	23.30 ± 1.54aA	23.80 ± 2.31aAB
Acidity _(in 21 day)_		24.80 ± 1.31aA	25.20 ± 1.06aA	25.50 ± 1.32aA	25.90 ± 1.15aA
WHC _(in 1 day)_		10.39 ± 0.53bD	8.81 ± 0.12cD	9.87 ± 0.20bD	11.16 ± 0.46aB
WHC _(in 7 day)_		30.86 ± 1.99bB	18.71 ± 0.48aC	32.82 ± 1.04bC	50.55 ± 0.92aA
WHC _(in 14 day)_		26.23 ± 1.08cC	26.09 ± 0.86cB	39.50 ± 0.71bB	51.62 ± 1.40aA
WHC _(in 21day)_		33.92 ± 0.82bA	30.41 ± 1.02cA	51.32 ± 0.43aA	50.65 ± 1.08aA

1. Data (mean ± standard deviation) are from three replications.

2. In the TQPI fortified desserts means in the same row with different lowercase letters (a–c) and in the same column with different uppercase letters (A–D) among dessert samples differ significantly (*p* ≤ .05).

3. Titicaca protein isolated (TQPI); Values are expressed as mean ± SD; dw: Dry weight,

^a^ Total carbohydrate (g/100 g) = 100− (m _fat_ ± m _ash_ ± m _proteins_).

^b^ Energy =4× (% protein ± %carbohydrates) ± 9× (% fat).

^c^ (g/100 g as fed) was unit of measurement.

^d^ (g/100 g dw) was unit of measurement.

^e^ (kcal/100 g dw) was unit of measurement.

The energy value obtained with the TQPI‐enriched dessert was higher than that of the control dessert (*p* ≤ .05), which may be related to the high‐fat content of the dessert. Similar results have already been achieved by researchers who fortified desserts with cumin and caraway flowers. Desserts fortified with 5% TQPI had the highest protein and dry matter content among samples. The fat content decreased significantly in 3% and 5% TQPI‐fortified samples. One of the possible reasons for this reduction can be related to TQPI addition in the formulation. It causes the ratio of milk (milk component) as a solid form to the total material (dry matter) decreased.

### 3.2 Titratable acidity and pH

The effects of TQPI content on the pH and acidity of low‐fat dairy desserts during the storage at 4°C are shown in Table 1. A significant decrease in pH was observed in all samples with increasing the storage time. The pH value of 5% TQPI‐fortified dessert was slightly lower than those of the others. It may be due to the presence of TQPI (with pH = 4.84 ± 0.03) in this dessert. Among the other samples, the pH value of TQPI desserts decreased with the TQPI concentration increased. The change in pH over the storage time at 4°C was similar to those observed by Chavan et al. (2010) and Kaur and Goswami (2020) (Chavan et al., 2010; Kaur & Goswami, 2020). The acidity of all TQPI‐fortified desserts increased by increasing the storage period. The highest acidity value belonged to 5% TQPI samples during the storage. Increment in TQPI levels in dessert may increase the acidity of dessert and reduce pH throughout the storage period. In this research, TQPI had acidity (16.33 ± 0.53°D) that had not influenced the acidity of fortified desserts. In the same research, Granato et al. (2010) found that desserts enriched with soy protein and guava juice contained organic acids. The organic acid had a significant effect on the acidity and pH of the product (Granato et al., 2010). Statistical analysis showed no significant interaction between the variables “TQPI %* time” (F9,32 = 0.75; *p* > .05) and (F9,32 = 0.15; *p* > .05) for pH and acidity, respectively. Therefore, the evolution of pH and acidity during the storage time might depend on the TQPI %.

### 3.3 Water‐holding capacity

Table 1 shows the change in WHC during 21 days of the storage. The WHC for all desserts increased during the storage. It is due to dry matter increased during the storage (Table 1) (Ünal et al., 2003). Desserts fortified with 5% TQPI had the highest WHC content. On the other hand, control desserts showed the lowest consistency which leads to lower WHC (*p* ≤ .05) compared to other desserts. Other studies have reported that the addition of protein results in finer networks, denser cross‐links, smaller pores, less shedding, and increased WHC (Amatayakul et al., 2006). Moreover, since heat is used in the dessert production process, it can affect the texture of the product and its syneresis. Whey protein contains intramolecular disulfide bridges that stabilize its structure. The sulfhydryl groups of β‐lactoglobulins are activated during the denaturation of proteins, the process tends to the formation of sulfhydryl disulfide interactions which occur between themselves and other proteins. As a result of those reactions, the rheological properties of coagulated milk gels seemed to affected by whey protein (Isleten & Karagul‐Yuceer., 2006). Additionally, there were significant differences for the interaction term “time*TQPI %” in the WHC values (F9,32 = 141.29; *p* ≤ .05), indicating that the evolution of WHC values in the dessert with time might depend on the applied TQPI %.

### 3.4 Amino acid profile analysis of dessert

The amino acid profile of TQPI‐fortified desserts is presented in Table 2. Tryptophan (1.87–2.87 g/100 g) is a vital amino acid, which is taking part in protein synthesis and functions as a precursor of biologically active components such as serotonin, melatonin, quinolinic acid, kynurenic acid, tryptamine, and coenzyme (Kałużna‐Czaplińska et al., 2019). Glutamic acid (0.41–1.26 g/100 g) may be significant for human health because glutamic acid is essential for the normal functioning of the human body as a protein ingredient and neurotransmitter (Asif et al., 2021). In the same research on dark chocolate fortified with QPI, the addition of quinoa has been observed to increase the number of amino acids, especially some essential amino acids (Schumacher et al., 2010). But protein insertion has limitations. The limitations of adding protein–herbal components are sensory characteristics, color, and leading to pH reduction at the levels of 5.42 ± 0.2 and 5.76 ± 0.28 in 3% and 5% TQPI‐fortified samples (Grek et al., 2018).

**TABLE 2:** Amino acid profile of fortified dessert with TQPI

Amino acid (g/100 g)	Fortified dessert with TQPI			
	Control	1%	3%	5%
Aspartic acid	0.19	0.41	0.47	0.52
Glutamic acid	0.41	0.83	1.08	1.26
Serine	0.31	0.40	0.44	0.48
Tyrosine	0.28	0.41	0.48	0.51
Arginine	0.39	0.46	0.49	0.58
Methionine	0.39	0.37	0.42	0.49
Tryptophan	1.87	2.28	2.68	2.87
Valine	0.22	0.30	0.33	0.46
Isoleucine	0.22	0.25	0.29	0.41
Lysine	0.61	0.57	0.60	0.81
Phenylalanine	0.33	0.36	0.43	0.51
Leucine	0.34	0.47	0.56	0.68
Histidine	0.53	0.51	0.59	0.68
Glycine	0.28	0.33	0.32	0.37
Threonine	0.23	0.39	0.47	0.54
Alanine	0.21	0.34	0.31	0.33

1. Data are from one replication.

*Titicaca quinoa* protein isolated (TQPI).

### 3.5 Color parameters

According to the statistical analysis, there were significant differences for the interaction “time*TQPI %” corresponding to the color parameter measurements that consist of L* (F3,40 = 1.98; *p* > .05), a* (F3,40 = 6.0; *p* ≤ .05), and b* (F3,40 = 5.03; *p* ≤ .05), indicating that the change in dessert color with the storage time might depend on the applied TQPI %, except L*. The data presented in Table 3 show the color attributes of the fortified desserts. Regarding the surface color, it was clear that the addition of TQPI decreased the color characteristics (L* and a*) as TQPI levels increased but the redness (b*) value increased. These results also showed that the highest increase in redness (a*) was found in dessert samples fortified with TQPI at levels 5% from the control. These results were in agreement with those reported by Mohamed et al. (2014) and Friedeck et al. (2003) who reported that L* and b* parameters decreased during the storage time (Friedeck et al., 2003; Mohamed et al., 2014). Drake et al. (2000) also reported that the brightness value (L*) of soybean yogurt was lower than that of milk yogurt. In this study, brightness (L*) and redness values (a*) decreased over time for desserts enhanced with a TQPI of 1%–5%.

**TABLE 3:** Color parameters of fortified dessert with TQPI

Color parameters	Day	Control	1%	3%	5%
L*	1	55.50 ± 1.29aA	39.50 ± 0.58bC	37.25 ± 2.06bB	27.00 ± 4.16cA
	7	54.75 ± 2.36aA	46.25 ± 3.86bB	34.00 ± 1.41cB	27.50 ± 1.29cA
	14	56.50 ± 4.65aA	49.75 ± 1.50aAB	36.75 ± 3.50cB	24.75 ± 1.50dAB
	21	59.25 ± 1.71aA	53.00 ± 2.16bA	43.00 ± 1.83cA	21.75 ± 1.50 dB
a*	1	0.75 ± 0.5aA	0.50 ± 0.57bA	−1.50 ± 1.29bA	−1.25 ± 0.95aA
	7	−0.50 ± 1.0abB	−0.75 ± 0.5bB	−1.25 ± 0.5bA	−1.75 ± 0.5aA
	14	−0.50 ± 0.05aB	−0.75 ± 1.5abB	−1.50 ± 0.57abA	−2.25 ± 0.5bAB
	21	−0.75 ± 0.5aB	−1.00 ± 0.81aB	−1.50 ± 1.5aA	−3.25 ± 0.95 bB
b*	1	9.00 ± 0.82aC	12.75 ± 2.5bC	18.75 ± 5.25aA	18.00 ± 1.83aB
	7	16.25 ± 2.22aB	17.25 ± 1.26aB	18.25 ± 1.50aA	17.75 ± 1.26aB
	14	17.50 ± 1.29bBA	20.25 ± 0.96aA	18.75 ± 0.96baA	18.5 ± 0.58bB
	21	19.25 ± 0.50bA	20.00 ± 0.82baA	20.25 ± 0.50baA	20.75 ± 0.96aA

1‐ Data (mean ± standard deviation) are from three replications.

2‐ Means in the same row with different lowercase letters (a–c) and means in the same column with different uppercase letters (A–C) among dessert samples differ significantly (*p* ≤ .05).

*Titicaca quinoa* protein isolated (TQPI).

### 3.6 Texture analysis

There were significant differences for the interaction “time*TQPI %” corresponding to the texture parameter measurements that consist of hardness (F3,16 = 177.71; *p* ≤ .05); adhesiveness (F3,16 = 9.0; *p* ≤ .05); cohesiveness (F3,16 = 42; *p* ≤ .05); springiness (F3,16 = 5; *p* ≤ .05); gumminess (F3,16 = 94; *p* ≤ .05); and chewiness (F3,16 = 6; *p* ≤ .05), indicating that the change in dessert texture parameters with the storage time might depend on the applied TQPI %. As indicated in Table 4, hardness, adhesiveness, springiness, gumminess, and chewiness increased but cohesiveness and springiness decreased as TQPI increased in the samples and at the end of the storage time for each sample separately. Because TQPI (>80% protein) was used in this research, it is likely that the differences in texture parameters among the dessert samples were attributable to the highest protein levels in the TQPI‐fortified desserts. Similar comments were made by a sensory panelist of yogurt containing a 5% soy protein concentrate. In general, increased protein content results in increased hardness and water‐holding capacity (WHC) due to the large number of proteins participating in the protein network. During dessert preparation, the use of heat in the presence of milk base and protein may cause the unfolding of whey proteins and denature them irreversibly. In these conditions, whey protein eventually aggregates with themselves and with casein (Lesme et al., 2020). Syneresis corresponds to serum release from the gel matrix that influences the textural properties of yogurts (Delİkanli Kiyak & Özcan, 2014)**.**

**TABLE 4:** Texture parameters of TQPI dessert

Texture parameters	Day	Control	1%	3%	5%
Hardness (g)	1	18.43 ± 0.6f	37.8 ± 1.01c	50.4 ± 0.85b	53.50 ± 0.92a
	21	24.93 ± 1.51e	53.43 ± 0.81c	57.73 ± 2.10	63.23 ± 1.46d
Adhesiveness (mJ)	1	0.35 ± 0.01d	0.74 ± 0.01c	1.10 ± 0.10a	1.32 ± 0.29ab
	21	0.63 ± 0.12c	0.67 ± 0.02c	0.65 ± 0.06c	0.88 ± 0.19bc
Cohesiveness	1	1.04 ± 0.01a	0.57 ± 0.03b	0.56 ± 0.02b	0.44 ± 0.04c
	21	0.55 ± 0.04b	0.61 ± 0.02b	0.64 ± 0.06b	0.64 ± 0.14b
Springiness (mm)	1	8.34 ± 0.12a	7.32 ± 0.14b	7.61 ± 0.39b	7.78 ± 0.41b
	21	6.00 ± 0.22c	5.84 ± 0.49c	5.08 ± 0.09d	5.00 ± 0.13d
Gumminess (g)	1	17.77 ± 0.15 h	20.32 ± 0.35 g	27.70 ± 0.70f	49.09 ± 1.17e
	21	52.49 ± 1.32d	55.27 ± 1.36c	60.70 ± 0.79b	67.30 ± 1.41a
Chewiness (mJ)	1	1.44 ± 0.04a	1.45 ± 0.23b	2.05 ± 0.16b	3.67 ± 0.26b
	21	7.96 ± 0.16c	8.41 ± 0.16c	9.87 ± 0.53b	11.41 ± 0.46a

1‐ Data (mean ± standard deviation) are from three replications.

2‐ Means with different lowercase letters in the same column and row (a–h) among dessert samples differ significantly (*p* ≤ .05).

3. *Titicaca quinoa* protein isolated (TQPI).

### 3.7 SEM

**FIGURE 1:**
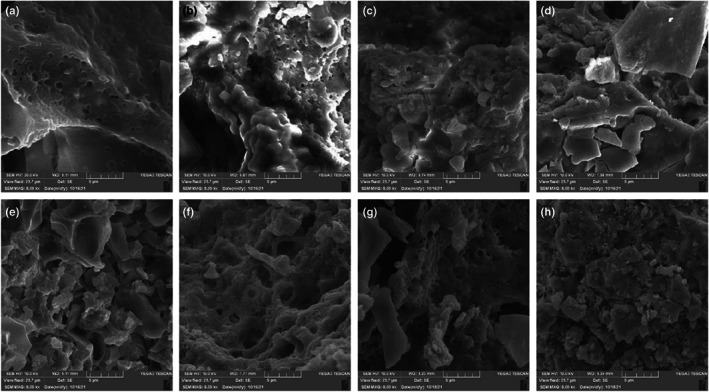
Scan electron microscopy images of TQPI fortified dessert samples. (a) Control; (b)1% TQPI; (c) 3% TQPI; (d)5% TQPI in first day of production, (e) Control; (f)1% TQPI; (g) 3% TQPI; (h)5% TQPI in 21th day of production

The SEM micrographs were obtained from desserts fortified with different concentrations of TQPI (Figure 1). They showed that the composition of the dessert gel was influenced by the TQPI concentration. A denser structure and fewer cavities and pores were observed when TQPI concentration increased as compared to the control. The reason for this finding may be related to the contribution of TQPI to enhance the formation of linking between the protein elements. These results were supported by increased WHC during the storage time in this study. These results could be compared with those of Remeuf et al. (2003) who observed that WPC contributed to urge the level of bridging between protein elements in yogurt (Remeuf et al., 2003). Moreover, the addition of TQPI also increased the levels of the total solid (TS) of fortified desserts. Interactions between the milk proteins were increased due to the enhancement of milk total solids and the formation of a stronger gel (Puvanenthiran et al., 2014). This increase can cause the formation of gel networks through cross‐linking during the milk fermentation. Similarly, Akalin et al. (2012) observed that as the ratio of whey protein increased, the networks have become finer, the size of the aggregate decreased, the interconnected networks have become denser, and the pores have become smaller (Akalin et al., 2012).

### 3.8 Sensory properties

**FIGURE 2:**
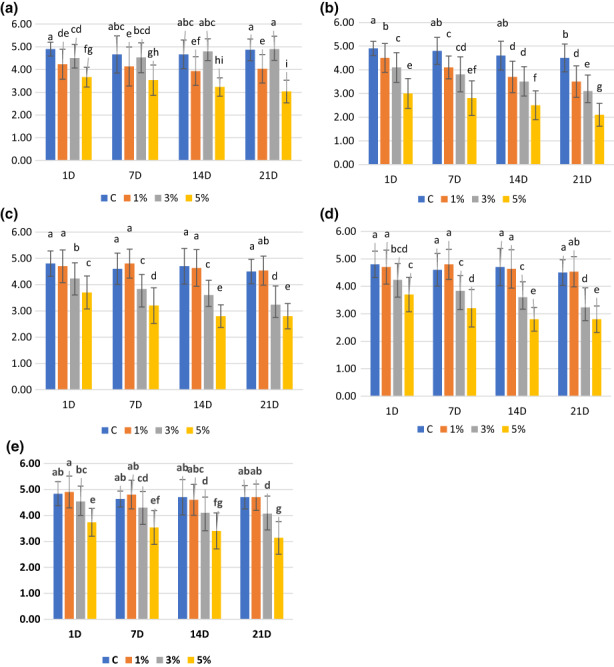
Sensory attributes of dessert samples supplemented with Titicaca quinoa protein isolated. Odor (a); color (b); taste (c); texture(d); total acceptability(e)

There were significant differences for the interaction “time*TQPI %” corresponding to the sensory parameter measurements that consist of color (F9,464 = 1.39; *p* ≤ .05); flavor (F_9,464_ = 5.31; *p* ≤ .05); odor (F9,464 = 3.0; *p* ≤ .05); texture (F9,464 = 3.0; *p* ≤ .05); and total acceptability (F9,464 = 1.08; *p* > .05), indicating that the change in dessert texture parameters with the storage time might depend on the applied TQPI %, except of total acceptability. Differences in the flavor, odor, texture, color, and total acceptability of samples in each desert were observed during the storage (Figure 2). Among the samples, by increasing the amount of TQPI, the amount of color, odor, taste, texture, and total acceptability components decreased. However, no significant difference was observed between C and 1% TQPI samples in taste, texture, and total acceptability score.

The odor score was almost constant during the storage period, except for the 5% of TQPI sample. A significant decrease was seen in the 5% TQPI sample at the end of the storage time. The taste, texture, and the total acceptability scores of C and the 1% samples were constant during the storage period, but the 3% and 5% TQPI sample scores nearly decreased. The color score decreased during the storage period in all samples. Among the samples, the lowest color score belonged to the 5% TQPI sample on the 21st day.

The green/grassy and sandy or coarse textures were detected in the 5% and 3% TQPI desserts. These results are in agreement with Shaviklo et al. (2011) who reported that one of the most objectionable defects in soy protein‐fortified ice cream which can be detected easily and affect texture was green/grassy and sandy. Repeated homogenization can reduce this problem (Shaviklo et al., 2011). The sensory analysis also revealed that the TQPI‐fortified desserts exhibited highest thickness/texture and were darkest in color, which is in agreement with instrumental analysis. These results of this study are in agreement with Friedeck et al. (2003) (Friedeck et al., 2003). Moreover, a different color was noticed for desserts containing 5% added TQPI by both sensory measurements and instrumental analysis, indicating that desserts with TQPI were less white in comparison to that of a typical low‐fat dessert. These changes may be related to Maillard browning during the storage that caused an increase in dark color in all samples during the storage time (Drake et al., 2000).

## 4 CONCLUSION

From the results of this study, we can conclude as follows that the incorporation of TQPI in low‐fat dairy desserts increased the acidity and decreased the pH of low‐fat dairy desserts over the storage period. The acidity slightly increased and pH decreased as the TQPI level increased in the dessert at the end of the storage time. TQPI, in high concentration (5 g/100 g), decreased the sensory score of the fortified dessert during the storage period. Moreover, the change in dessert sensory parameters with the storage time might depend on the applied TQPI %, except for the total acceptability. In this regard, desserts supplemented with TQPI have proven to be an excellent source of protein, including abundant essential amino acids, especially glutamic acid and tryptophan. The change in dessert texture, color, and WHC parameters with the storage time might depend on the applied TQPI %, except for pH, acidity, and L* color parameter. From the results, we can conclude that adding up to 5 g/100 g of TQPI to the dessert increased the protein content, acidity, b*, hardness, adhesiveness, springiness, gumminess, chewiness, and WHC. However, L*, a*, cohesiveness, springiness, and pH were decreased. TQPI‐fortified desserts exhibited highest thickness/texture and were darkest in color. Electron microscopy provided the formation evidence of a strong network in a TQPI‐fortified dessert, while the control dessert had a loose network. Thus, TQPI supplementation could be a potential option to produce high‐quality, low‐cost, and low‐fat desserts with enhanced nutritional and technological properties.

## FUNDING INFORMATION

This research did not receive any specific grant from funding agencies in the public, commercial, or not‐for‐profit sectors.

## CONFLICT OF INTEREST

The authors declare that they do not have any conflict of interest.

## ETHICAL APPROVAL

This study does not involve any human or animal testing

## INFORMED CONSENT

Written informed consent was obtained from all study participants

## Data Availability

Data available on request from the authors
